# Students’ Satisfaction with a Web-Based Pharmacy Program in a Re-Regulated Pharmacy Market

**DOI:** 10.3390/pharmacy5030047

**Published:** 2017-08-25

**Authors:** Maria Gustafsson, Sofia Mattsson, Gisselle Gallego

**Affiliations:** 1 Department of Pharmacology and Clinical Neuroscience, Umeå University, SE-90187 Umeå, Sweden; sofia.mattsson@umu.se; 2 School of Medicine, The University of Notre Dame, New South Wales 2010, Australia; gisselle.gallego@nd.edu.au

**Keywords:** pharmacy education, web-based, student satisfaction, re-regulation

## Abstract

In response to the shortage of pharmacists in Northern Sweden, a web-based Bachelor of Science in Pharmacy program was established at Umeå University in 2003. In 2009, the Swedish pharmacy market was re-regulated from a state monopoly to an open market, but it is unknown what impact this has had on education satisfaction. The objectives of this study were to examine the level of satisfaction among graduates from a web-based pharmacy program and to describe what subjects and skills students would have liked more or less of in their education. A secondary objective was to compare the level of satisfaction before and after the Swedish pharmacy market was re-regulated. A cross-sectional survey was conducted in 2015 with all alumni who had graduated from the pharmacy program between 2006 and 2014 (*n* = 511), and responses to questions about graduates’ satisfaction with the program were analyzed (*n* = 200). Most graduates (88%) agreed or strongly agreed that the knowledge and skills acquired during their education were useful in their current job. The graduates stated that they would have wanted more applied pharmacy practice and self-care counselling, and fewer social pharmacy and histology courses. Further, 82% stated that they would start the same degree program if they were to choose again today, and 92% agreed or strongly agreed that they would recommend the program to a prospective student. Graduates were more likely to recommend the program after the re-regulation (*p* = 0.007). In conclusion, pharmacy graduates were very satisfied with their education, and no negative effects of the re-regulation could be observed on program satisfaction.

## 1. Introduction

Education satisfaction has been suggested to be an important influence on students’ successful learning [1]. After completing their degree, graduates need to find their roles when practicing pharmacy. Discrepancies between expectations of job assignments and actual job assignments have been found in previous studies among pharmacists, and this might affect both education and job satisfaction [2,3].

In 2003, a web-based three-year Bachelor of Science in Pharmacy program was introduced at Umeå University. The program was initiated in response to the expressed request of Apoteket AB (the state-owned pharmacy monopoly at the time) because of their difficulties in recruiting pharmacists in the inland parts of Northern Sweden. The pharmacy program at Umeå University is web-based, and the education is mostly conducted through a virtual learning environment with regular online meetings between students and teachers and some mandatory meetings on campus in Umeå. During the on-campus meetings, the students engage in activities such as laboratory work, oral presentations, and role-play. The virtual learning environment contains recorded lectures, assignments, animations, and references to the literature. During the online meetings, the students and teachers discuss different topics concerning the specific course. The structure and content of the program has generally been the same over the years, besides regular revisions to keep the course material up to date. The courses constituting the program can be divided into three main areas—pharmaceutical chemistry, biomedical sciences, and pharmacy. After completing the bachelor’s degree program, the students are eligible to apply to the Master of Pharmaceutical Science program in order to obtain a master’s degree. This master’s program was introduced at Umeå University in 2010.

In Sweden, the main place of work for graduates from the Bachelor of Science in Pharmacy program is at community pharmacies [4]. In 2009, the Swedish pharmacy market changed from a state-owned pharmacy monopoly (Apoteket AB) to an open pharmacy market in what is called the “re-regulation” of the Swedish market [5,6,7]. Because of the re-regulation, by the end of 2010 about 200 additional community pharmacies had opened across Sweden [4]. As a result, there was an increased demand for pharmacists [8,9,10], especially in areas of the country where there was a shortage of skilled personnel, such as Northern Sweden [11]. However, in 2011 the market appeared to become saturated, and some pharmacies announced redundancies and pharmacies were closed in response to poor financial results. Since the re-regulation, community pharmacies have worked on a retail supply model that focuses on the high throughput of prescriptions and the sale of non-prescription products [6,12]. As a consequence, new tasks have emerged for pharmacists, although the overall responsibilities are the same before and after the re-regulation. These new tasks might affect the skills and knowledge needed for pharmacists, and consequently the education needed to prepare them for these tasks.

Little is known about education satisfaction among pharmacy programs in Sweden. Hence, the main aim of this study was to examine the level of education satisfaction among pharmacy graduates at Umeå University and to describe what subjects and skills students would have liked more or less of in their education. A secondary objective was to compare the level of satisfaction before and after the re-regulation of the Swedish pharmacy market.

## 2. Materials and Methods

### 2.1. Survey Instrument

The study questionnaire was developed using information from the literature [13] as well as general student surveys from other departments within the university. The questionnaire consisted of 35 questions divided into the following five sections: (1) employment characteristics; (2) job satisfaction; (3) satisfaction with the education; (4) demographics; and (5) open-ended questions where the graduates were asked to provide further comments. The data used for this study contains information from Section 3 and Section 4.

In Section 3, graduates were asked to express agreement or disagreement with two statements using a five-point Likert scale (1 = “strongly disagree” to 5 = “strongly agree”): (1) “The knowledge and skills you acquired during your training are useful in your current job” and (2) “I would recommend the program to a prospective student”. Graduates were asked to respond to the question “If you had to choose today, would you start the same degree program?” using a five-point Likert scale (1 = No, I would not have started any education at all to 5 = Yes). Graduates were also asked if they believed their duties at work reflected their education (Yes/No). Those who chose “No” were asked to provide an explanation (open question). Graduates were also asked if they work in a sector that is completely outside their field. Those who chose “Yes” were asked why they chose to change careers after obtaining their pharmacy degree. In order to further explore how the knowledge and skills acquired during the education are useful in their current job, students were also asked two open-ended questions: “What in your education would you have liked more of?” and “What in your education would you have liked less of?” The responses to these questions were then categorized based on frequency. Only categories with more than 10 responses were included. The graduates could include subjects within the program as well as other subjects and skills (e.g., communication, laboratory work). Section 4 asked demographics questions concerning gender, age, income, marital status, and area of living.

### 2.2. Data Collection

A paper copy of the questionnaire was posted in May 2015 to people who graduated from the Bachelor of Science in Pharmacy program (a three-year web-based program) and/or the Master of Pharmaceutical Science program (a two-year web-based program) between 2006 and 2014 and who had a Swedish address in the university’s administrative system (*n* = 437). The paper copy also had a link to an online version. To protect graduates’ privacy, address labels were printed by the administrative department, and a research assistant was in charge of posting the envelopes. Those with no Swedish registered address (*n* = 74) were sent an email invitation asking if they wanted to participate or opt out from further communication regarding the survey. The contents of the paper version and the online version were identical. The link to the online version was active for two months (May and June 2015).

### 2.3. Data Analysis

Those who graduated before (2006–2009) and after (2010–2014) the re-regulation and those who were satisfied and not satisfied with their education were compared using the Pearson chi-square test and Student’s *t*-test for dichotomous and continuous variables, respectively. Education satisfaction was measured with the response to the statement “I would recommend the program to a prospective student“. The variables were dichotomized into “disagree” (Likert responses 1–3) and “agree” (Likert responses 4 and 5). Job satisfaction was measured with the question “All things considered, how often are you satisfied with your job?” and the answers were dichotomized into “not satisfied” (those who responded “never or rarely” and “sometimes satisfied”) and “satisfied” (those who responded “satisfied most of the time” and “all of the time”).

The answers to the questions regarding educational satisfaction among those who graduated before the re-regulation (2006–2009) and after the re-regulation (2010–2014) were compared, and a multivariate logistic regression model was constructed. The model had educational satisfaction as the dependent variable and included age and year of graduation (2006–2009 or 2010–2014) as independent variables. In the multivariate model, the variables were dichotomized into “disagree” (Likert responses 1–3) and “agree” (Likert responses 4 and 5) and into “no” (Likert responses 1–4) and “yes” (Likert response 5). In the analysis comparing year of graduation, 192 persons were included in the final analysis because data for eight respondents were missing. 

Responses were collected and analyzed using SPSS version 23. A *p*-value of <0.05 was considered statistically significant. Open-ended responses were analyzed using a modified thematic analysis, which involved an open coding technique.

### 2.4. Ethics

No ethical committee approval was sought prior to beginning this research because it is not obligatory under Swedish law for this type of study. All data were anonymized before analysis. The participants in this study were informed about de-identification of the material and about the aim of the study, and they consented to the data being used for research purposes.

## 3. Results

Of 511 graduates, 222 graduates completed the survey for a response rate of 43%. In this study, only graduates from the Bachelor of Pharmaceutical Science program were analyzed, leaving a final sample of 200 graduates. Respondents graduating from the Master of Pharmaceutical Science program (*n* = 21) were excluded because this paper focuses on the education satisfaction among graduates from the bachelor program. This also includes graduates with both a bachelor and master’s degree (*n* = 9). One respondent did not state the degree they received and was also excluded from further analysis. The characteristics of the respondents are shown in Table 1. The majority of respondents were female (96%), the mean age was 40.1 years, and most were employed at a community pharmacy (85%). The mean years in their current position was 3.7 years, and the majority (92%) were satisfied with their jobs. Respondents who graduated in 2006–2009 were older (43.0 years) compared to those who graduated in 2010–2014 (37.1 years), and there was a significant difference between years in current position of 5.2 years vs. 2.3 years. Among those who worked in a community pharmacy, most graduates were employed at Apoteket AB, and this was true for both groups. There was no statistically significant difference in job satisfaction between the two groups.

The majority of graduates (88%) agreed or strongly agreed that the knowledge and skills acquired during the education were useful in their current job. The majority of the graduates (92%) agreed or strongly agreed that they would recommend the program to a prospective student (Table 2). Further, 82% stated that if they were to choose today, they would start the same pharmacy program. Of the graduates, 2.6% stated that they would have rather studied pharmacy at another university. A few graduates (8.3%) stated that they would have rather started another education program at the same university, and 2.1% would have chosen another educational area at a different university. Finally, 5.2% of the graduates would not have commenced any university studies at all if they were to choose today.

There were no differences among graduates who were satisfied with their education and graduates who were not satisfied regarding age and gender (Table 3). Significantly more graduates among those who were satisfied with their education were also satisfied with their job (*p* < 0.001).

Graduates reported that during their education they would have liked more of subjects such as applied pharmacy practice and self-care counselling and more training in skills such as communication. Other subjects mentioned included business administration, economics, sales, and leadership. Further, they wanted less of subjects such as social pharmacy and histology (Table 4).

The majority of the participants (75%) thought that their duties at work reflected their education. Graduates who answered “no” to this question were asked to provide further comments. From the analysis of these open-ended responses, two broad themes were identified: (1) I learned about this but have not had any use of this knowledge, and (2) This was not included in the program, but I need it at work.

Theme 1: I learned about this but have not had any use of this knowledge.

Some of the graduates explained that they have more knowledge than they need in their current job, which is exemplified by this comment:

“*The program gives much deeper and more detailed knowledge than what is needed in order to handle prescriptions at a pharmacy*”.

Another graduate mentioned:

“*Everything you learn about, for example, pharmacodynamics, pharmacokinetics and general chemistry, you unfortunately never use at a community pharmacy… This knowledge, that you knew really well, is forgotten… Very sad*”.

These comments highlight how some of the knowledge that the graduates gained might not be used and/or applied in their current jobs. In addition, some respondents mentioned that they would like to use their knowledge more, and they also felt that the knowledge they have is not requested, as exemplified by this quote:

“*The boring thing is that when you work at a community pharmacy there are few people who ask for our competence. Neither employers nor customers are especially interested in what we know*”.

A common reason for the mismatch between their education and their current job was that some graduates perceived that the current focus of community pharmacies is on selling products. One of the graduates noted:

“*Now it is only about selling products (outpatient care products) and earning money. You rarely get to use the knowledge you have. For example, giving advice and informing/discussing medicines with the customers. There is no time for that*”.

Theme 2: This was not included in the program, but I need it at work.

Some of the graduates mentioned that some subjects within the pharmacy degree were missing. For example, they would have liked more practical knowledge in areas such as patient counselling and therapeutics, but less focus on chemistry and basic science, as exemplified by these quotes:

“*I think the program focused too much and in too much detail on chemistry at the expense of, for example, outpatient care and animal medicines, which we had on only one occasion*”.

“*More practice, more about medicines and how they work in the body. More depth and a longer course*”.

Some respondents also mentioned competences outside the field of pharmacy, and particularly emphasized the lack of education regarding sales during the program; however, they did not say if they wanted more emphasis on knowledge of “how to sell”.

“*There is a lot of focus on sales at pharmacies, but we never heard anything about that in the program*”.

Another graduate mentioned:

“*During my studies I did not learn to sell cosmetics and body lotion, which is something I am expected to do at work*”.

Graduates were also asked if they work in a sector that is completely outside of pharmacy, and nine (4.5%) answered yes to this question. These graduates were asked why they chose to change careers after obtaining their pharmacy degree. Most of the explanations provided were about job satisfaction. One of the graduates commented: “It’s just stress. There are no opportunities for continuing professional development, and it is difficult to get a good working schedule.” Another mentioned: “Working at a pharmacy did not live up to my expectations. I thought it was like working in any other store”.

Two of the graduates mentioned that they could not get a permanent job in their hometown.

“*I worked at a pharmacy for six months, had children, and then got a better job in my hometown in my previous profession*”.

In order to explore if graduates’ satisfaction with their education had changed after the re-regulation, a regression analysis was performed. The analysis showed that a higher proportion of the respondents who graduated in 2010–2014 would recommend the program to a prospective student compared to respondents who graduated in 2006–2009 (*p* = 0.007) (Table 5). No other statistically significant differences regarding education satisfaction after the re-regulation were found in this analysis.

## 4. Discussion

The main findings from this study are that pharmacy graduates were very satisfied with their education. Also, graduates stated that during their education they would have wanted more subjects such as applied pharmacy practice and self-care counselling, and fewer social pharmacy and histology courses. Further, people graduating after the re-regulation were more likely to recommend the program to a prospective student. That the vast majority of respondents were female and the mean age was 40 years is similar to what has been reported for the Swedish pharmacy workforce [7,13].

This study provided an opportunity for graduates to look back at their education and to consider if it provided the necessary skills and knowledge to perform their job. Most of the respondents stated that if they were to choose today, they would start the same degree program. Comparisons with other studies are somewhat difficult because different designs and questions are used; however, compared to one study from Australia, the results in the present study indicate a higher education satisfaction [14]. Further, a recent investigation from another university in Sweden found that almost half of the respondents three years after graduation from a Master of Science in Pharmacy program were not satisfied with their choice of education and would not choose the same education again [15]. In Sweden, there are two different professional degrees within pharmacy—prescriptionists (with a bachelor’s degree) and pharmacists (with a master’s degree). Both professional degrees have the same rights to dispense drugs in a community pharmacy. However, while prescriptionists mainly work in community pharmacies, pharmacists also work in other areas such as the pharmaceutical industry, universities, hospitals, and the government. The difference in education satisfaction between bachelor and master graduates could be due to a discrepancy in the students’ expectations regarding their professional career. Because of the rather favorable labor market in community pharmacy at present, graduates with a master’s degree often work there but might have a desire to work elsewhere within the pharmaceutical area, e.g., the pharmaceutical industry or a hospital pharmacy.

Because community pharmacy is the major workplace for the graduates, it was important to understand whether the graduates’ education had responded to changes in the labor market. Graduates were positive about the knowledge and skills acquired and were willing to recommend the program to a prospective student. It is interesting to note how this is even higher after the re-regulation. Despite the changes in the pharmacy market, graduates would be willing to study pharmacy again. This might indicate that although the pharmacy market and the role of the pharmacist have changed, the education still succeeds in preparing the students for relevant job assignments. Another possible explanation is the increased number of community pharmacies with different owners [5], which has expanded the labor market and perhaps provided a greater variation regarding job assignments. The majority of the graduates thought that their duties at work reflected their education. However, one-third of the graduates considered that their knowledge was not being used and that they did things they had little or no training for. Business administration, economics, sales, and leadership were subjects mentioned by the graduates as missing in their education, subjects that might be of more importance for today’s pharmacists compared to before the re-regulation. This is a reflection of how practice has become more commercial and how the vital source of revenue in community pharmacies has moved towards selling non-drug articles (for example, cosmetics and vitamins) [12]. Some graduates also commented on how their knowledge is not recognized, which could be due to a discrepancy between what community pharmacists do and what they want to do. This is found in both Nordic studies [2,3] as well as in Swedish settings [7].

Graduates in the present study wanted more pharmacy practice, self-care counselling, and communication training. This is probably a reflection of their duties at a community pharmacy. Usual work tasks at a community pharmacy are to serve at the counter or in the OTC self-selection department, advising customers [16]. A pharmacist should help patients choose the most effective, safe, and convenient pharmacotherapeutic option, and to be able to do this communication is important. Research has shown that improved communication can improve both satisfaction and adherence to prescribed therapy among patients [17,18].

Although the majority of the graduates work at community pharmacies, graduates from the Bachelor of Science in Pharmacy program can also work in other places such as the pharmaceutical industry and government agencies. Offering broad knowledge as well as expertise that addresses the labor market changes is a balancing act. There is a coexistence of different perspectives that needs to be considered when developing and changing higher education, and perhaps one way is to allow more dialogs in an external context.

The results of this study show that the re-regulation did not have a great impact on graduates’ education satisfaction. Perhaps the education is broad enough to address the new labor market. It seems that Umeå University pharmacy graduates are satisfied with their education, but not with some of the tasks at their workplace.

One of the limitations of this study was that all graduates completing the survey are now working in a liberalized pharmacy market, and graduates’ opinions about their education might be reflected by their current workplace, i.e., in the re-regulated pharmacy market. Further, some of the graduates might have experience with the former market, but the extent of this is not known. Also, years of work experience have not been corrected for, which might be a confounder. It would have been interesting to ask the graduates more detailed questions about how they have experienced the re-regulation in order to be able to compare the situation before and after, but this was beyond the scope of the study. There is also a risk of selection bias, and it cannot be excluded that students who are more positive about their education were more likely to complete the survey, which might influence the results.

## 5. Conclusions

This study provided an opportunity to investigate education satisfaction among graduates between 2006 and 2014. Pharmacy graduates were very satisfied with their education, and no negative effects of the re-regulation could be observed on program satisfaction. The subjects and skills the students would have liked more or less of in their education probably reflected their current duties at the community pharmacy.

## Figures and Tables

**Table 1:** Characteristics of the participants.

	Total Sample	Graduated 2006–2009	Graduated 2010–2014	*p*-Value
Total number of people *n*	200	96 *	96 *	
Women *n* (%)	191 (95.5)	96 (100)	88 (91.7)	0.004
Age mean ± SD	40.1 ± 8.8	43.0 ± 8.5	37.1 ± 8.6	<0.001
Current employment				
Community pharmacy *n* (%)	169 (84.5)	76 (79.2)	86 (89.6)	
Hospital pharmacy *n* (%)	5 (2.5)	3 (3.1)	2 (2.1)	
Pharmaceutical company *n* (%)	5 (2.5)	3 (3.1)	2 (2.1)	
County council *n* (%)	4 (2.0)	1 (1.0)	3 (3.1)	
Municipality *n* (%)	4 (2.0)	4 (4.2)	0 (0.0)	
Dose-dispensing pharmacy *n* (%)	3 (1.5)	2 (2.1)	0 (0.0)	
Others** *n* (%)	10 (5.0)	7 (7.3)	3 (3.1)	
Pharmacy				
Apoteket AB *n* (%)	45 (22.5)	23 (24.0)	20 (20.8)	
Kronans Apotek *n* (%)	40 (20.0)	21 (21.9)	18 (18.8)	
Apoteket Hjärtat *n* (%)	39 (19.5)	20 (20.8)	18 (18.8)	
Years in current position	3.7 ± 2.7	5.2 ± 2.7	2.3 ± 1.9	<0.001
All things considered, how satisfied are you with your job?				0.306
Satisfied *n* (%)	173 (91.5)	85 (89.5)	88 (93.6)	
Not satisfied *n* (%)	16 (8.5)	10 (10.5)	6 (6.4)	

* Data regarding graduation year for eight respondents was missing; Others** include drug product manufacturing, international clinical testing, medical technology, goods control at head office, teaching pharmaceutical technicians in training, university, and various work places.

**Table 2:** Graduates’ satisfaction with their education.

	Percentage of Graduates (%)				
	Strongly Disagree	Disagree	Neither Agree or Disagree	Agree	Strongly Agree
The knowledge and skills acquired during the education are useful in my current job (*n* = 217)	1.5%	5.2%	5.1%	**61.4%**	26.9%
I would recommend the program to a prospective student (*n* = 209)	2.0%	3.6%	2.6%	28.6	**63.3**%

**Table 3:** Comparison between graduates satisfied and not satisfied with their education.

Total Number of People	Graduates Satisfied with Their Education *n* = 180	Graduates Not Satisfied with Their Education *n* = 16	*p*-Value
Age	40.0 ± 9.0	37.9 ± 7.6	0.294
Women *n* (%)	172 (95.6)	15 (93.8)	0.742
All things considered, how satisfied are you with your job?			<0.001
Satisfied	170 (95.5)	8 (53.3)	
Not satisfied	8 (4.5)	7 (46.7)	

**Table 4:** What subjects and skills graduates would like to have more or less of in their education.

Graduates *n* (%)	*n* = 200
What graduates would have liked more of in their education	
Applied pharmacy practice *n* (%)	35 (17.5)
Communication *n* (%)	25 (12.5)
Drug interactions *n* (%)	11 (5.5)
Other subjects within pharmacy * *n* (%)	34 (17.0)
Pathology *n* (%)	17 (8.5)
Pharmacology *n* (%)	23 (11.5)
Pharmacotherapy *n* (%)	12 (6.0)
Self-care counselling *n* (%)	30 (15.0)
Subjects not within pharmacy ** *n* (%)	12 (6.0)
What the graduates would have liked less of in their education	
Chemistry *n* (%)	14 (7.0)
Histology *n* (%)	24 (12.0)
Social pharmacy *n* (%)	16 (8.0)
Statistics *n* (%)	12 (6.0)

* Includes: clinical pharmacy, drug knowledge, adverse reactions, legislation, good manufacturing practice, generic substitution, pharmacokinetics, pharmacodynamics, anatomy, cytostatics, extemporaneous compounding, therapy recommendations, drugs and the elderly, skin care, drug evaluation, dose assessment, parallel imported drugs, contraindications, drug dispensing, toxicology, microbiology, and pharmaceutics. ** Includes: sales, promotion, business administration, economics, psychology, behavioral science, crisis management, and work environment.

**Table 5:** Multivariate logistic regression including different questions regarding education satisfaction.

Total Number of People	Graduated before the Re-Regulation 2006–2009	Graduated after the Re-Regulation 2010–2014	Odds Ratio *	95% Confidence Interval	*p*-Value
The knowledge and skills acquired during the education are useful in my current job *n* (%)	81/95 (85.3)	86/94 (91.5)	2.437	0.901–6.588	0.079
I would recommend the program to a prospective student *n* (%)	82/94 (87.2)	91/94(96.8)	6.945	1.171–28.149	0.007
The duties at work reflect the education *n* (%)	70/95 (73.7)	76/96 (79.2)	1.851	0.888–3.860	0.100
If I had to choose today, I would start the same degree program *n* (%)	70/90 (77.8)	80/94 (85.1)	2.065	0.912–4.674	0.082

* Controlled for age.
